# Immediate Effect of Balance Taping Using Kinesiology Tape on Dynamic and Static Balance after Ankle Muscle Fatigue

**DOI:** 10.3390/healthcare8020162

**Published:** 2020-06-09

**Authors:** Hyun-Su Choi, Jung-Hoon Lee

**Affiliations:** 1 Department of Biomedical Health Science, Graduate School, Dong-Eui University, Busan 47340, Korea; nicecleanday@hanmail.net; 2 Department of Physical Therapy, College of Nursing, Healthcare Sciences and Human Ecology, Dong-Eui University, Busan 47340, Korea; 3 Integrated Physical Medicine Institute, Dong-Eui University, Busan 47340, Korea

**Keywords:** balance, balance taping, kinesiology tape, fatigue, ankle

## Abstract

The objective of this study was to investigate whether ankle balance taping (ABT) applied after muscle fatigue-inducing exercise can cause immediate improvements in dynamic and static balance. A total of 31 adults (16 males and 15 females) met the inclusion criteria. The experiment was designed using a single-blinded, randomized controlled trial. Changes in static and dynamic balance were measured before and after inducing muscle fatigue in the ankles and after ABT or ankle placebo taping (APT). After ankle muscle fatigue-inducing exercise, both the ABT and APT groups showed significant increases in surface area ellipses in the static state with eyes open (*p <* 0.05), and significant increases in surface area ellipses in the static and dynamic states with eyes closed (both *p <* 0.05). After taping of the fatigued ankle, surface area ellipses decreased significantly when eyes were open and closed in the static and dynamic states, but only in the ABT group (*p <* 0.05). Static balance was significantly different between groups (eyes open, 36.2 ± 86; eyes closed, 22.9 ± 46.7). Dynamic balance was significantly different between groups (eyes open, 68.6 ± 152.1; eyes closed, 235.8 ± 317.6). ABT may help prevent ankle injuries in individuals who experience muscle fatigue around the ankles after sports and daily activities.

## 1. Introduction

Muscle fatigue is caused by repeated muscle contractions during high-intensity exercise or activities over a prolonged time, and it reduces the ability of the muscles to generate force due to temporary reduction in the ability to contract [1,2]. Muscle fatigue can impair the performance of the joints and the body during voluntary movement [3], and has a negative effect on the ability to control balance due to low reactivity to stimulation and impairment of joint proprioception and kinesthesis [4,5].

The ability to maintain balance under static or dynamic situations is a prerequisite for performing various activities of daily living [6,7]. Ankle muscles play a central role in maintaining balance in one-leg and two-leg standing positions [8,9], but when muscle fatigue occurs, the joints become unstable, causing increased postural sway and decreased ability to maintain balance [10]. Therefore, muscle fatigue near the ankle joint may increase the risk of ankle sprain injury [11].

Kinesiology taping is often used in clinical practice for treating musculoskeletal and nervous disorders, and its use in sports for preventing and treating sports-related injuries is also increasing. Previous studies have reported that kinesiology taping improves kinesthesis and proprioception [12,13,14], pain control and muscle strength [15,16], and joint range of motion (ROM) [13,17]. A recent study reported that ankle balance taping (ABT) using kinesiology tape showed immediate improvements in the dynamic balance of young soccer players with ankle instability [18] and the static balance of healthy adults [19].

There are very few studies on the immediate effects of ABT with kinesiology tape on dynamic and static balance following ankle muscle fatigue. Therefore, the objective of this study was to investigate whether ABT with kinesiology tape applied after muscle fatigue-inducing exercise affects the dynamic and static balance abilities of individuals with no ankle pain or instability.

## 2. Material and Methods

### 2.1. Participants

To calculate the sample size needed, G-Power 3.1 (University of Dusseldorf, Dusseldorf, Germany) was used with the significance level (alpha level) set at 0.05, statistical power at 0.8, and effect size at 0.7 based on the anticipated differences in static balance between pretest and post-intervention [20]. The calculation indicated that 24 participants were needed. Thirty-one adult participants who did not have any ankle pain or instability provided their consent to participate in the study. This study was approved by the Institutional Review Board of Dong-eui University (DIRB-201602-HR-E-011). 

The exclusion criteria for the study were as follows: (1) orthopedic or neurosurgical injury in the past 6 months (history of surgery without experiencing sprain or fracture in the ankles or lower extremity); (2) use of any medication in the past 3 months; (3) ankle edema; (4) abnormality of the nervous system, including the vestibular organ and cerebellum; and (5) past history of contact dermatitis. 

### 2.2. Study Design

**Figure 1:**
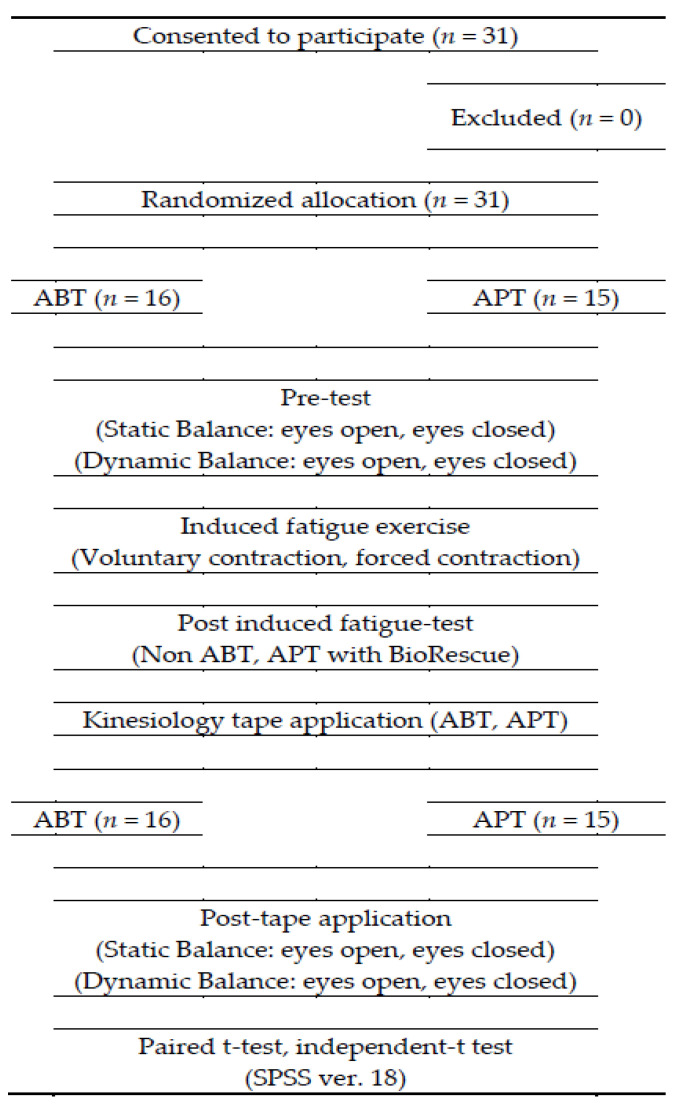
Flow diagram for the study.

This study was designed as a single-blinded, randomized controlled trial. Participants were randomly assigned to either the control group (APT) or experimental group (ABT). All measurements were carried out by the same examiner, in a university laboratory, who was stationed behind a nontransparent screen and was blinded to group allocation. The order of measurement (eyes open, eyes closed, static, and dynamic) was randomized. The randomization process was carried out using a computer-generated table of random numbers. Taping was performed by a physical therapist with more than 10 years of experience. First, the static and dynamic balance of both groups were measured with eyes open and then with eyes closed, respectively. Immediately after the ankle muscle fatigue-inducing exercise, measurements of static and dynamic balance were measured again. ABT with kinesiology tape or APT was applied immediately and static and dynamic balance were measured for a third time. The flowchart of this study is shown in Figure 1.

### 2.3. Measurement

**Figure 2:**
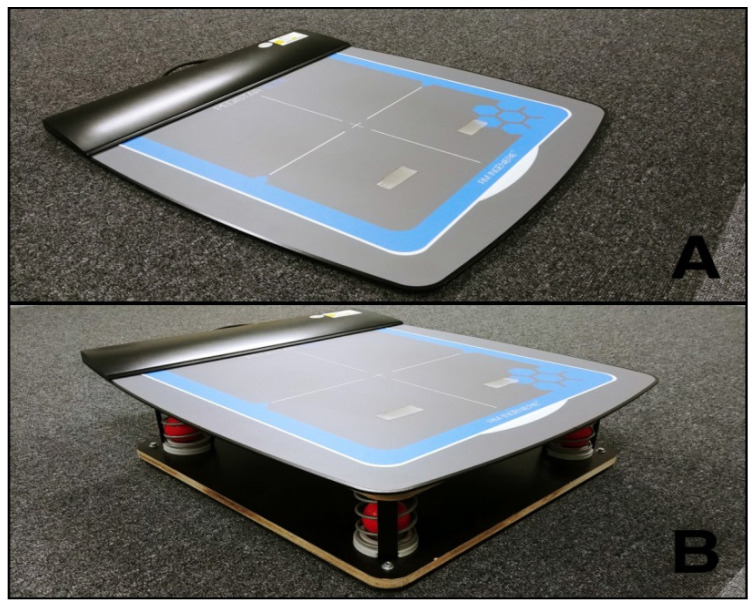
BioRescue plate for static standing balance (**A**); BioRescue plate for dynamic standing balance (**B**).

The study used BioRescue (RM Ingenierie, Rodez, France) for the measurement of changes in static and dynamic balance before and after inducing muscle fatigue in the ankles and after applying kinesiology tape. BioRescue (RM Ingenierie) is a device with 1600 sensors connected to a foot plate (610 × 580 × 10 mm) that measures the balance ability of the participant based on changes in the surface area ellipse of the center of pressure (mm^2^) (Figure 2) [21]. A previous study has demonstrated high test–retest reliability of this device (intraclass correlation coefficients = 0.83 − 0.95) [22].

### 2.4. Fatigue Protocol

The method suggested by Gefen et al. [11] which consists of voluntary contraction and forced contraction, was used to induce ankle muscle fatigue. For voluntary contraction, 40 repetitions of dorsiflexion and plantarflexion were performed with the participant seated in a chair without the feet touching the ground. For forced contraction, the participant performed 25 repetitions of lifting the heels as much as possible in plantarflexion and maintaining that position for 1 s before returning the heels to the ground in a standing position whilst placing their hands on the back of a chair support structure.

### 2.5. Intervention

#### 2.5.1. Ankle Balance Taping (ABT)

**Figure 3:**
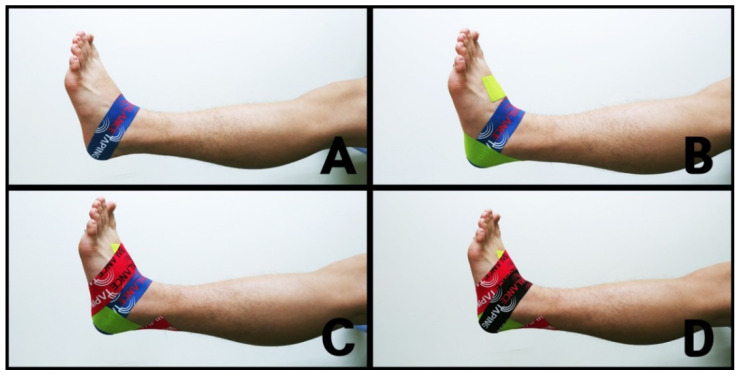
Ankle balance taping (ABT): (**A**) taping was applied from the dorsal center of the talus bone to the calcaneus bone; (**B**) taping was applied from 5 cm above the medial malleolus and passed through the lateral calcaneus below the subtalar joint; (**C**) taping was applied from 5 cm above the lateral malleolus and passed through the medial calcaneus below the subtalar joint; (**D**) taping was reapplied on top of the area covered by the initial taping.

With the participants seated comfortably, the kinesiology tape (BB TAPE, WETAPE Inc., Paju, Korea) was stretched by approximately 30–40% and applied to both ankles, taking care not to stretch the origin and insertion (approximately 2–3 cm) in order to protect the skin [19]. The ABT application method was consisted of four steps [23]: (1) while maintaining slight ankle dorsiflexion, taping was applied to both sides, starting from the dorsal center of the talus bone to the calcaneus bone (Figure 3A); (2) while maintaining ankle inversion, taping was applied from 5 cm above the medial malleolus and passed through the lateral calcaneus below the subtalar joint, and then directed towards the lateral aspect of the dorsum (Figure 3B); (3) while maintaining ankle eversion, taping was applied from 5 cm above the lateral malleolus and passed through the medial calcaneus below the subtalar joint, and then directed towards the medial aspect of the dorsum (Figure 3C); and (4) while maintaining slight ankle dorsiflexion, taping was reapplied on top of the area taped by the initial taping to strengthen ankle support (Figure 3D) [18,19,23,24].

#### 2.5.2. Ankle Placebo Taping (APT)

**Figure 4:**
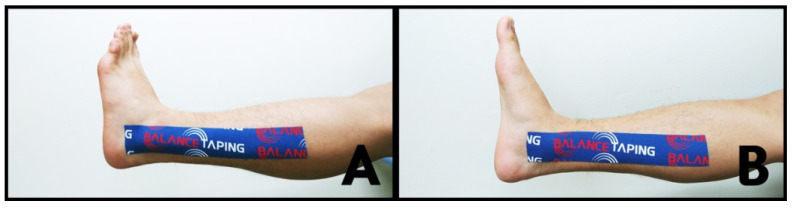
Ankle placebo taping (APT): (**A**) taping was applied from below the medial malleolus to half of the area below the inner knee; (**B**) taping was applied from below the lateral malleolus to half of the area below the outer knee.

With the participant seated comfortably, the kinesiology tape was stretched by approximately 30%–40% and applied to both ankles, without stretching the origin and insertion sites (approximately 2–3 cm) in order to protect the skin [25]. APT consisted of two steps: (1) taping was applied from below the medial malleolus to half of the area below the inner knee (Figure 4A) and (2) from below the lateral malleolus to half of the area below the outer knee (Figure 4B) [18,19,24].

### 2.6. Statistical Analysis

The data were analyzed using SPSS 18.0 (IBM Corp., Armonk, NY, USA). The Shapiro–Wilk test was used to test for normality. Independent sample *t*-tests were used to identify significant differences in the baseline static and dynamic balance values in the ABT and APT groups, while paired samples *t*-tests were performed for comparisons of static and dynamic balance before and after ankle muscle fatigue-inducing exercise. Paired samples *t*-tests were also performed for comparisons of changes in static and dynamic balance after ABT and APT application following muscle fatigue-inducing exercise. The significance level (α) was set to 0.05.

## 3. Results

### 3.1. Participants’ General Characteristics

The general characteristics of the study participants are provided in Table 1. There were no significant differences in baseline static and dynamic balance in the ABT and APT groups (*p >* 0.05) (Table 2).

### 3.2. Static Balance and Dynamic Balance with Eyes Open

After ankle muscle fatigue-inducing exercises, both the ABT and APT groups showed a significant increase in surface area ellipses in the static state with eyes open (*p <* 0.05) (Table 3). After ankle muscle fatigue-inducing exercise, neither group showed significant changes in surface area ellipses in the dynamic state with eyes open (*p >* 0.05) (Table 3).

After taping of the fatigued ankle, surface area ellipses in the static state with eyes open decreased significantly only in the ABT group (*p <* 0.05) (Table 4). After taping of the fatigued ankle, surface area ellipses in the dynamic state with eyes open decreased significantly only in the ABT group (*p <* 0.05) (Table 4).

### 3.3. Static Balance and Dynamic Balance with Eyes Closed

After ankle muscle fatigue-inducing exercise, both the ABT and APT groups showed significant increases in surface area ellipses in the static state with eyes closed (*p <* 0.05) (Table 5). After ankle muscle fatigue-inducing exercise, both the ABT and APT groups showed a significant increase in surface area ellipses in the dynamic state with eyes closed (*p <* 0.05) (Table 5).

After taping of the fatigued ankle, surface area ellipses in the static state with eyes closed decreased significantly only in the ABT group (*p <* 0.05) (Table 6). After taping of the fatigued ankle, surface area ellipses in the dynamic state with eyes closed decreased significantly only in the ABT group (*p <* 0.05) (Table 6).

## 4. Discussion

Results demonstrate that both ABT and APT groups showed a significant increase in surface area ellipses in the static state with both eyes open and eyes closed after muscle fatigue-inducing exercise. Both groups showed a significant increase in surface area ellipses in the dynamic state with eyes closed after muscle fatigue-inducing exercise. However, in the dynamic state with eyes open, there were no significant differences in surface area ellipses after fatigue-inducing exercise.

Muscle fatigue may cause proprioceptive and kinesthetic impairment in joints [4]. Muscle fatigue may impair the maintenance of the joints and body during voluntary movement and cause low reactivity to stimuli [3]. Mello et al. [5] reported that muscle fatigue may have a negative effect on the ability to maintain standing posture and control balance due to reduced performance of the neuromuscular control mechanism. Therefore, surface area ellipses may increase significantly after muscle fatigue-inducing exercise due to impaired proprioception and kinesthetic sense and reduced neuromuscular control.

Vision has a major impact on postural stability, regardless of age [25]. It provides essential sensory information for maintaining dynamic stability during exercise in humans [26,27,28,29]. In the present study, surface area ellipses did not increase significantly after muscle fatigue-inducing exercise in the dynamic state with eyes open, and this may be due to the assistance of visual feedback on an unstable surface.

Compared to the APT group, the ABT group showed a significant decrease in surface area ellipses in both the static and dynamic states after muscle fatigue-inducing exercise. In a recent study by Lee and Lee, [18] balance ability was improved by applying ABT to a functionally unstable ankle caused by injury during a soccer match. The elasticity of kinesiology tape that returns to its original length may help return the joint to its original position [30]. The elasticity of kinesiology tape for ABT may help the ankle joint to return more quickly to an ankle’s neutral position on an unstable surface [16,19]. When kinesiology tape that has been stretched by 30–40% is applied to the ankles, the tape is instantly stretched even further by the ankle’s movement, and joint stability can be increased by the elasticity of the tape quickly returning the ankle’s neutral position [18,19].

This study had the following limitations. Firstly, since the intervention was applied to healthy adults with no pathological symptoms, the generalizability of the findings may be limited. Secondly, we did not use isokinetic equipment that induced muscle fatigue according to the muscle characteristics of each individual or provided evidence of ankle fatigue in this study. Thirdly, there may have been a learning effect for the measurement of post-fatigue activity that could have contributed to the results, since participants were younger and fatigue may not have been severe enough to induce changes. Fourthly, participants were not completely blinded to the interventions. Fifthly, we did not investigate and compare the use of tape directly applied to the muscle, such as the tibialis anterior and calf muscle, near the ankle where muscle fatigue was induced. In the future, additional studies should be conducted to improve on these limitations.

## 5. Conclusions

Following ankle muscle fatigue-inducing exercise, which reduced static and dynamic balance abilities, ABT improved static and dynamic balance with eyes open and closed. Therefore, we suggest that ABT helps recover immediately decreased balance resulting from muscle fatigue after daily physical activities and sports activities, thereby preventing ankle injuries.

## Figures and Tables

**Table 1:** General characteristics of the study participants in each group (n = 31).

Variables	ABT (n = 16)	APT (n = 15)	*p*
Sex (male/female) (%)	8 (50%)/8 (50%)	8 (53.3%)/7 (46.7%)	0.86
Age (years)	23.3 ± 2.6	22.0 ± 2.0	0.13
Height (cm)	167.4 ± 10.4	169.3 ± 7.7	0.56
Weight (kg)	61.9 ± 11.9	64.1 ± 10.1	0.60

Data are expressed as numbers or mean ± standard deviation; ABT, ankle balance taping; APT, ankle placebo taping.

**Table 2:** Comparison of initial surface area ellipse values in the two groups.

	Surface Area Ellipse (mm^2^)		
Variables	ABT	APT	*p*
Static, EO	34.5 ± 10.7	35.4 ± 24.0	0.90
Dynamic, EO	211.0 ± 125.4	179.2 ± 217.0	0.62
Static, EC	30.9 ± 17.6	23.1 ± 11.0	0.15
Dynamic, EC	540.2 ± 399.1	541.7 ± 73.8	0.99

Data are expressed as numbers or mean ± standard deviation; ABT, ankle balance taping; APT, ankle placebo taping; EO, eyes open; EC, eyes closed.

**Table 3:** Comparison of variations in surface area ellipses on a static and a dynamic plate with eyes open before and after fatigue-inducing exercise.

		Surface Area Ellipse (mm^2^)		
Plate	Variables	Pre-exercise	Post-exercise	*p*
Static	ABT	34.5 ± 10.7	110.9 ± 69.3	0.01 *
	APT	35.4 ± 24.0	109.2 ± 94.3	0.01 *
Dynamic	ABT	211.0 ± 125.4	318.7 ± 397.1	0.26
	APT	179.2 ± 217.0	329.6 ± 395.0	0.13

Data are expressed as numbers or mean ± standard deviation; ABT, ankle balance taping; APT, ankle placebo taping; * *p* < 0.05.

**Table 4:** Comparison of variations in surface area ellipses on a static and a dynamic plate with eyes open after fatigue-inducing exercise and after taping.

		Surface Area Ellipse (mm^2^)		
Plate	Variables	Post-exercise	Post-taping	*p*
Static	ABT	110.9 ± 69.3	28.1 ± 19.8	0.01 *
	APT	109.2 ± 94.3	64.3 ± 105.8	0.26
Dynamic	ABT	318.7 ± 397.1	114.0 ± 37.9	0.04 *
	APT	329.6 ± 395.0	182.6 ± 190.0	0.15

Data are expressed as numbers or mean ± standard deviation; ABT, ankle balance taping; APT, ankle placebo taping, * *p* < 0.05.

**Table 5:** Comparison of variations in surface area ellipses on a static and a dynamic plate with eyes closed before and after fatigue-inducing exercise.

		Surface Area Ellipse (mm^2^)		
Plate	Variables	Pre-exercise	Post-exercise	*p*
Static	ABT	30.9 ± 17.6	58.4 ± 35.8	0.01 *
	APT	23.1 ± 11.0	58.2 ± 39.7	0.01 *
Dynamic	ABT	540.2 ± 399.1	806.1 ± 506.4	0.01 *
	APT	541.7 ± 73.8	777.0 ± 204.1	0.01 *

Data are expressed as numbers or mean ± standard deviation; ABT, ankle balance taping; APT, ankle placebo taping, * *p* < 0.05.

**Table 6:** Comparison of variations in surface area ellipses on a static and a dynamic plate with eyes closed after fatigue-inducing exercise and after taping.

		Surface Area Ellipse (mm^2^)		
Plate	Variables	Post-exercise	Post-taping	*p*
Static	ABT	58.4 ± 35.8	20.4 ± 19.1	0.01 *
	APT	58.2 ± 39.7	43.3 ± 65.8	0.51
Dynamic	ABT	806.1 ± 506.4	327.9 ± 124.9	0.01 *
	APT	777.0 ± 204.1	563.7 ± 442.5	0.08

Data are expressed as numbers or mean ± standard deviation; ABT: ankle balance taping; APT: ankle placebo taping, * *p* < 0.05.
